# 399. High Burden of Invasive Fungal Infections in Critical Care Units of Bangladesh: Findings from Ongoing sentinel Surveillance

**DOI:** 10.1093/ofid/ofaf695.137

**Published:** 2026-01-11

**Authors:** Tanzir Ahmed Shuvo, Nusrat Jahan Shaly, Farhat Jaby Pammi, Md Kamrul Islam, Adiba Tasnim, Shams e Tabriz, Fahmida Dil Farzana, Mohammad Abdul Aleem, Ismet Nigar, Sanjoy kumer dey, Md Abdul Mannan, Montosh Kumar Mondal, Tahsinul Amin, Jesmin Akter, Dilruba Ahmed, Debashis Sen, Asifa Kumkum, Sazzad Bin Shahid, Mahbuba Chowdhury, Fahmida Chowdhury, Sayeeda Huq

**Affiliations:** icddr,b, Dhaka, Dhaka, Bangladesh; icddr,b, Dhaka, Dhaka, Bangladesh; icddr,b, Dhaka, Dhaka, Bangladesh; icddr,b, Dhaka, Dhaka, Bangladesh; icddr,b, Dhaka, Dhaka, Bangladesh; icddr,b, Dhaka, Dhaka, Bangladesh; icddr,b, Dhaka, Dhaka, Bangladesh; icddr,b, Dhaka, Dhaka, Bangladesh; Bangladesh Medical University, Dhaka, Dhaka, Bangladesh; Bangladesh Medical University, Dhaka, Dhaka, Bangladesh; Bangladesh Medical University, Dhaka, Dhaka, Bangladesh; Bangladesh Medical University, Dhaka, Dhaka, Bangladesh; Dhaka Medical College and Hospital, Dhaka, Dhaka, Bangladesh; Dhaka Medical College Hospital, Dhaka, Dhaka, Bangladesh; icddr,b, Dhaka, Dhaka, Bangladesh; icddr,b, Dhaka, Dhaka, Bangladesh; icddr,b, Dhaka, Dhaka, Bangladesh; Dhaka Medical College Hospital, Dhaka, Dhaka, Bangladesh; Dhaka Medical College Hospital, Dhaka, Dhaka, Bangladesh; icddr,b, Dhaka, Dhaka, Bangladesh; icddr,b, Dhaka, Dhaka, Bangladesh

## Abstract

**Background:**

Invasive fungal infections (IFIs) are associated with severe complications and high mortality, particularly in critically ill patients. Despite their clinical significance, the true burden of IFIs remains poorly understood in low- and middle-income countries (LMICs) like Bangladesh, primarily due to the lack of structured surveillance systems. This study aims to assess the burden of IFIs among patients admitted to critical care units in Bangladesh.

**Figure:**
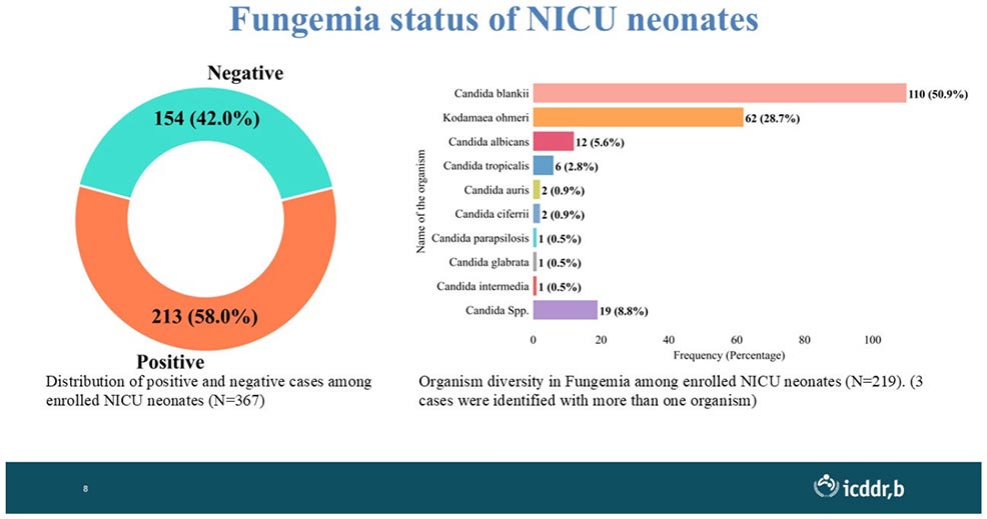
Circulating fungal pathogens among neonates

**Figure:**
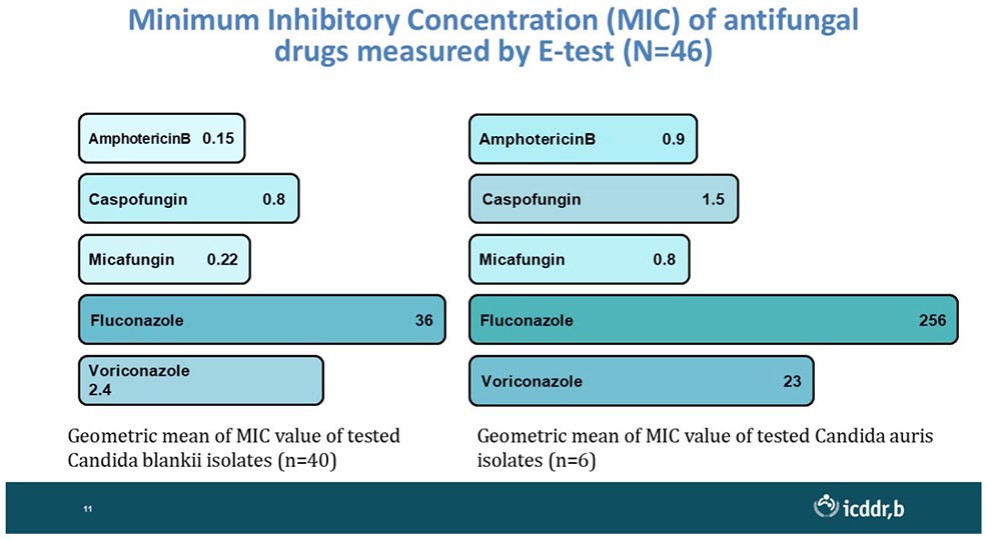
MIC values of different antifungal for Candida auris and Candida blankii isolates

**Methods:**

Since August 2022, hospital-based sentinel surveillance has been ongoing in the ICU, NICU, and PICU of two tertiary-level hospitals in Dhaka. By March 2025, a total of 829 patients with suspected IFIs were enrolled based on predefined criteria. Blood and endotracheal aspirate samples were processed using microscopy, culture, and identification via Vitek 2 and Vitek MS systems. Antifungal susceptibility testing (AFST) was performed using E-test methods for selected isolates.

**Results:**

Overall, 35% of the enrolled patients had fungal-positive blood cultures, though positivity varied by unit. Adult ICUs showed 7.5% positivity (n=351), with *Candida auris* and *Candida tropicalis* (6 each) being predominant. PICUs demonstrated 31% positivity (n=123), mostly infected with *Candida blankii* (18). NICUs revealed the highest burden (60%, n=355), with *C. blankii* (107) and *Kodamaea ohmeri* (61) as predominant species. Empirical antifungal use, particularly fluconazole and voriconazole, was common (26%); however, high azole resistance was noted, especially in *C. auris* and *C. blankii* and was associated with higher mortality.

**Conclusion:**

There is significant IFI burden in critical care units of Bangladesh with frequent identification of emerging and resistant pathogens. These findings highlight the urgent need for affordable diagnostics, standardized AFST protocols, and incorporation of IFI-specific strategies into antimicrobial stewardship programs to ensure rational anti-fungal use and curb resistance.

**Disclosures:**

All Authors: No reported disclosures

